# Supplementary material for: Are carbon emissions trading and green financial instruments synergistic? -Comprehensive quantitative research based on content analysis
Source: PLoS One. 2024 Mar 7;19(3):e0298601. doi: 10.1371/journal.pone.0298601 (PMC10919601; doi:10.1371/journal.pone.0298601)
Supplement: S1 Data — (ZIP) [file pone.0298601.s001.zip › Data/地级市Z_F工作报告.zip!/║╙─╧/─╧╤⌠╩╨/└┤╫╘╣1⁄2╓┌║┼í╛┬φ┐╦╩2╛▌═°í┐_2018.docx]

各位代表：

现在，我代表市人民政府，向大会报告工作，请予审议，并请各位政协委员和其他列席人员提出意见。

一、工作回顾

本届政府自2014年3月任职以来，面对复杂的外部环境和繁重的发展任务，在市委的坚强领导下，在市人大和市政协的监督支持下，坚持以习近平新时代中国特色社会主义思想为指导，主动适应经济发展新常态，全面落实中央和省委、省政府决策部署，统筹推进稳增长、促改革、调结构、惠民生、防风险各项工作,经济发展稳中有进，社会大局和谐稳定，人民群众安居乐业。

——综合实力稳步提升。2017年，全市生产总值达到3377.7亿元，年均增长8.2%。一般公共预算收入达到174.8亿元，年均增长9.1%。固定资产投资达到3733.2亿元，年均增长15.6%。三次产业结构持续优化，二三产业比重比2013年提高1.8个百分点。粮食产量稳定在120亿斤以上。

——城乡发展更加协调。中心城区、县城、重点乡镇和美丽乡村建设全面推进，城乡面貌明显改观，全市常住人口城镇化率达到44.7%、提高6.4个百分点；高起点规划建设南阳新城区，中心城区新建、续建城市道路29条，建成区面积达到155平方公里、人口达到160万人；实施百城建设提质工程，县城建设亮点纷呈；西峡县太平镇、镇平县石佛寺镇、邓州市穰东镇成为全国特色小镇，66个村成为全省美丽乡村试点，城乡一体化水平不断提升。

——发展后劲持续蓄积。改革开放全面深化，简政放权、商事制度改革等工作走在全省前列，市场主体达到38万户、增加19万户，稳居全省第3位；南阳卧龙综保区封关运行，京宛对口协作成效明显，中关村南阳科技园等一批项目落户南阳。创新驱动深入实施，高新技术企业达到88家，国家创新型试点城市通过考评验收。上市企业达到10家。老界岭、恐龙遗迹园成功创建国家5A级景区。

——基础支撑更加坚实。南水北调中线工程建成通水。宁西铁路二线建成通车，郑万高铁、蒙华铁路开工建设，宁西高铁、呼南高铁豫西通道争取工作取得重要进展。特别是经过多年来的不懈努力，郑万高铁将于明年开通，南阳即将进入“高铁时代”。内邓、三淅、邓鄂等高速公路建成通车，周南、栾西等高速公路开工建设，高速公路通车里程位居全省第一。南阳机场通航城市达到17个，年旅客吞吐量达到88万人次，机场迁建前期工作有序进行。内乡煤电运一体化、南召天池抽水蓄能电站等重大能源项目加快推进，风电、光伏发电装机规模达到94万千瓦，成为国家第一批创建新能源示范市。信息化建设成效明显，被确定为国家智慧城市试点、“宽带中国”示范市。

——生态环境明显好转。环境治理力度加大，蓝天白云明显增多，水体质量持续改善，南水北调中线水源地水质稳定达标；单位生产总值能耗下降18.6%。累计造林212万亩，森林覆盖率达到39.5%，被评定为全国绿化模范城市，跻身首批国家生态文明先行示范区。

——人民生活持续改善。财政用于民生支出累计达到1675亿元，年均增长10%。城乡居民人均可支配收入达到19119元，年均增长9.9%；居民消费价格涨幅稳定在2%以内。23.96万农村贫困人口稳定脱贫,贫困发生率由5.52%下降到3.21%。累计实现各类就业66.8万人,城镇登记失业率控制在4%以内。城乡教育均衡发展，“全面改薄”累计投入25亿元，强力推进中心城区15年一贯制完全学校建设五年计划（2017-2021），新建、改扩建中小学41所，普通中小学新增学位6.6万个。教育教学质量大幅提升，全市普通高考一本上线率、录取率连年攀升，3年有4名考生分别荣获全省高考文理科第一名，书写了我市教育史上浓墨重彩的一笔。医药卫生体制改革不断深化，优质医疗卫生资源逐步扩充，投资64亿元实施36个卫生基础设施项目，新晋升4家“三甲”医院。建设各类保障性住房5.8万套，改造农村危房9.8万户。公共文化设施基本实现全覆盖。全民健身活动扎实开展。

通过实践探索，我们走出了一条符合南阳实际、富有南阳特色的发展路子。特别是2017年，我们认真落实党的十九大精神，按照省委、省政府的决策部署，围绕建设重要区域中心城市目标定位，强力实施“两轮两翼”战略，重抓重推“九大专项”，加快推动经济高质量发展，全市经济社会发展呈现良好态势，各项工作取得新成绩。

（一）经济运行稳中有进

坚持搞活存量、优化增量、扩充总量、提升质量，经济运行稳中有进、转中向好。

1.运行总体平稳。坚持稳中求进工作总基调，以服务实体经济为导向，加强监测调度，狠抓薄弱环节，强化要素保障，积极化解风险，促进经济平稳运行。全市生产总值增长6.8%，固定资产投资增长9.9%，规模以上工业增加值增长6.1%，一般公共预算收入增长7.7%。

2.结构不断优化。打好转型发展攻坚战，深化供给侧结构性改革，产业转型升级步伐加快。服务业增加值增长9.2%，对经济增长的贡献率达到52.3%。高新技术产业增长19.8%，高成长性产业增长13.1%。种植业中经济作物面积占比提高7.4个百分点，新增绿色有机农产品基地17万亩；托管、流转耕地面积722万亩，新增家庭农场、农民专业合作社3388家。

3.效益持续提升。推动质量、效率、动力变革，努力培植财源，发展质量效益稳步提升。规上工业企业利润增长15.3%，高于上年10.9个百分点。13家企业进入省百强民营企业、上榜数量全省第二。税收收入增加4.4亿元。居民人均可支配收入增长9.7%，持续高于经济增速。

（二）发展动能持续增强

大力推进项目建设、科技创新、改革开放，不断蓄积发展后劲、激发内生动力、释放市场活力。

1.以项目建设蓄后劲。把项目建设作为实体经济发展的主抓手，309个省、市重点项目累计完成投资838亿元，占年度计划的146%，威奥斯图车辆减振器、海纳电子机器人、无人机高端信息产业园等“三个三十”重大项目完成投资340亿元，占年度计划的135%。67个项目进入省PPP项目库。争取国家储备林一期项目授信贷款108亿元。

2.以科技创新增动力。大力实施创新驱动发展战略，与省科技厅、省农科院建立会商机制，高新区争取列入郑洛新国家自创区辐射区取得新进展，南阳科技大市场挂牌运营。发放科技创新贷款贴息5000万元、科技创新券3740万元。新增高新技术企业26家，新增省级以上创新平台19家。新业态新模式蓬勃发展，电商交易额达到660亿元、增长69%。

3.以改革开放添活力。把改革开放作为加快转型跨越的关键，全面激发发展活力。强化改革推动。坚持“一跟进、两聚焦”，34项重点改革年度任务全面完成，城市综合执法局组建运行，农村产权制度改革、国企改革等重点改革事项有序推进，党政机关公务用车制度改革全面完成，科技创新券制度、生态文明工作机制、教育体制改革等得到上级肯定。实施开放带动。开放领域持续扩大，开放水平持续提升。综保区功能逐步完善，入驻企业21家。京宛合作持续深化，科教卫生、文化旅游、生态农业等方面合作全面加强，中关村领创空间、创业基地初见成效。成功举办玉雕节等招商引资活动，星凯龙新能源汽车等一批重大项目落地建设，实际利用外资5.87亿美元，实际利用省外资金626亿元。进出口总额131.2亿元、增长22.7%，其中出口总额108.2亿元、增长20%。

4.以现代金融活血脉。新引进保险机构4家，平安银行、中信信用卡、华林证券入驻南阳。社旗森霸光电成功上市。新增存贷比达到80.1%、提高37.7%。社会融资总规模达到249亿元。

（三）城乡建设力度加大

统筹实施中心城区振兴工程和乡村振兴战略，强力推进百城建设提质工程，城乡建设步入快车道。

1.中心城区加快发展。新城区规划建设全面启动，高铁片区建设全面展开，高速引线和7条道路开工建设，“一主两副”月季园等重点项目扎实推进。以“双创双建”为抓手，老城区改造提升步伐加快，卧龙岗武侯祠创建国家5A级景区、古宛城保护利用等重大工程启动实施，新建、改造市政道路12条，内河水系建设、棚户区改造等工作顺利推进。城市组团发展取得积极进展。

2.百城提质全面展开。坚持市县联动、全域推进，谋划项目1658个、计划总投资4017亿元，年度完成投资341亿元。统筹百城建设提质工程和文明城市创建，成功创建国家卫生城市，荣获全国未成年人思想道德建设工作先进城市，西峡县成功创建全国文明城市，火海救人英雄王锋同志荣获第六届全国道德模范，城市形象和市民素质明显提升。

3.美丽乡村持续推进。深入开展农村人居环境整治，垃圾处理长效机制逐步建立。完善农村基础设施，新解决7.13万贫困人口饮水安全问题，新建农村公路851公里，改造危桥7069延米。唐河县、新野县入选省美丽乡村示范县。创建和培育国家级、省级乡村旅游示范村24个。

（四）民生工作扎实有效

着力办好民生实事，加强社会治理创新，群众获得感、幸福感、安全感持续增强。

1.脱贫攻坚深入实施。坚持精准扶贫、精准脱贫基本方略，突出抓好产业扶贫、易地扶贫搬迁等关键环节，探索实施“千企帮千村”等扶贫新模式，11.83万贫困人口稳定脱贫，1.83万人完成易地扶贫搬迁。

2.环境治理成效明显。重拳整治大气污染，中心城区空气优良天数达到235天，比上年增加36天、多于全省11天。全面落实河长制，中心城区温凉河、汉城河、梅溪河、三里河综合治理初见成效，县域38条河道完成综合治理，10个国家考核断面水质平均达标率99%。积极创建国家森林城市，完成造林51万亩。

3.民生保障不断加强。在财力紧张的情况下，持续加大民生投入，财政用于民生的支出占比达到80.4%。新增各类就业14.4万人。在全省率先开通基本医疗保险、大病保险、大病补充保险“一站式”即时结算服务，群众看病就医更加方便。探索实施医保救助工程，困难群众门诊慢性病、重特大疾病门诊病种、住院报销比例分别提高25个百分点、14个百分点和10个百分点。“先诊疗、后付费”模式在全市全面铺开，有效降低了群众医药费用负担。谋划实施“政福保”工程。城镇保障房建设开工率100%，改造农村危房2.4万户。中心城区完全学校建设进展顺利。南阳师范学院获批硕士授权单位，南阳理工学院进入全国高校创新创业50强。

4.社会治理扎实有效。积极防范化解各类风险，反恐维稳有力有效，非法集资、房地产市场、农运会后续问题等风险化解持续深入。安全生产、食品药品安全、信访稳定、社会治安综合治理等工作平稳有序，荣获全省综治和平安建设优秀省辖市称号。

本届政府任职以来，我们自觉接受人大监督、政协民主监督和社会监督，认真办理人大代表建议和政协提案。加强民主法治建设，落实法治政府建设实施纲要，全面推进依法行政，不断强化行政监察和审计监督，政府自身建设和治理能力不断提升。国防动员、人民防空、双拥等工作持续深化，残疾人、妇女儿童、红十字等事业健康发展，统计、物价、供销、档案、史志、气象、地震、外事、侨务、民族宗教、机构编制、公积金管理、公共资源交易等工作都取得了新的成绩。

各位代表！南阳的变化有目共睹，南阳的发展鼓舞人心。成绩来之不易，奋斗充满艰辛。这是以习近平总书记为核心的党中央坚强领导的结果,是省委、省政府和市委正确领导的结果，是全市人民努力奋斗的结果。在此,我代表市人民政府，向全市广大工人、农民、知识分子，向驻宛人民解放军指战员、武警官兵、公安民警，向所有关心支持帮助南阳发展的各界朋友，表示崇高的敬意和衷心的感谢！

各位代表！在肯定成绩的同时，我们也清醒地认识到,南阳发展中还存在不少困难和问题：一是发展不平衡、不充分，产业结构不够优化，城乡发展不够协调，人均生产总值处于全省靠后位次，与洛阳、襄阳等地综合实力差距有拉大的趋势，建设重要区域中心城市任重而道远。二是增长动力不足，部分企业经营困难，民间投资乏力，创新能力不强，发展动能转换滞后。三是民生领域短板突出，脱贫攻坚任务艰巨，城乡居民收入偏低，公共服务体系不健全，农运会后续问题解决、房地产市场整治等工作推进缓慢，群众在就业、就学、医疗、养老等方面还有许多不满意的地方。四是防风险压力大，企业风险、金融风险不容忽视，反恐维稳、水质保护、安全生产等领域存在薄弱环节。五是政府职能转变还不到位，政务服务质量和效率与企业和群众的期盼有较大差距，一些政府工作人员能力素质不适应新时代要求，一些领域不正之风和腐败问题依然存在。我们将正视问题，认真解决，不负人民重托。

二、今后五年和2018年工作安排

今后五年，是决胜全面建成小康社会、开启全面建设社会主义现代化新征程的关键时期。市委六届四次全会和六次全会对标党的十九大精神，贯彻省委、省政府决策部署，明确了战略目标，描绘了发展蓝图，对全市上下以党的建设高质量推动经济发展高质量，加快建设重要区域中心城市，在中原更加出彩中展示新担当、新作为作了全面动员。

审视当前，展望未来，我们承担着推动高质量发展、决胜全面小康、建设重要区域中心城市的繁重任务，同时也面临着诸多有利条件：机遇难得,中央加大基础设施领域补短板的力度，实施积极的财政政策和稳健的货币政策，省委、省政府赋予南阳重要区域中心城市发展定位，只要我们加快融入“三区一群”建设，就可以借势借力、加快发展。优势明显，我们具有得天独厚的资源优势、区位优势、产业优势、市场优势、生态优势，正处于工业化、城镇化加速推进期，发展基础坚实、后劲充足、潜力巨大。态势向好，经过多年来的持续发展，南阳站上了新的历史起点，发展目标明确、思路清晰，弘扬了正气，凝聚了人心，锻炼了队伍，树立了形象，全市上下心齐气顺、人心思进，蓄势崛起呈现出光明前景。总的来看，我市仍处于大有可为的重要机遇期，我们要坚定信心、振作精神，加压增责、奋发作为，开创南阳高质量发展新局面，创造出无愧于时代、无愧于组织、无愧于人民的一流业绩。

今后五年政府工作的总体要求是：高举习近平新时代中国特色社会主义思想伟大旗帜，深入贯彻党的十九大精神和习近平总书记调研指导河南时的重要讲话精神，全面落实省委十届六次全会和市委六届六次全会精神，坚持新发展理念，坚持稳中求进工作总基调，突出“四个着力”，打好“四张牌”，推动县域治理“三起来”，做到乡镇工作“三结合”，全力打好“三大攻坚战”，围绕建设重要区域中心城市目标定位，强力实施“两轮两翼”战略，重抓重推“九大专项”，以党的建设高质量推动经济发展高质量，如期全面建成小康社会，开启新时代南阳全面建设社会主义现代化新征程，奋力谱写中原更加出彩的南阳篇章。

根据高质量发展的要求，结合市委六届四次全会提出的奋斗目标，建议今后五年经济社会发展的主要目标是：到2020年与全国一道全面建成小康社会，并乘势而上全面推进新时代社会主义现代化强市建设，高质量发展取得重大阶段性成效，重要区域中心城市建设取得重大突破，在全省发展大局中的地位和作用更加突显。

——综合实力显著提升。经济增速高于、发展质量优于全省平均水平，科技创新能力全面增强，单位生产总值税收贡献率明显提高，生产总值占全省比重稳定提升，在全国百强城市中位次有所前移。

——转型发展步伐加快。现代化经济体系加快构建，产业结构更加优化，高端制造、智能制造加速发展，战略新兴产业支撑有力，工业化、信息化基本实现，农业现代化取得明显进展，城镇化质量和水平大幅提升。

——环境质量更加优化。污染防治攻坚取得决定性成效，到2020年空气质量达到二级标准，地表水监测断面水质达到Ⅲ类以上，大气环境质量明显提升，水环境质量根本改善，土壤环境质量稳步好转，生态安全屏障基本建成，建成全国新能源示范城市、国家森林城市和国家生态文明先行示范区。

——人民生活全面改善。现行标准下农村贫困人口全部脱贫，贫困县全部摘帽。公共服务体系更加健全，社会事业全面发展，社会保障体系更加完善，城乡居民收入与地区生产总值同步增长。

——社会治理规范高效。社会文明程度明显提高，社会主义核心价值观深入人心，成功创建全国文明城市。法治南阳建设持续推进，社会治理社会化、法治化、智能化、专业化水平明显提高，彰显公平正义、共建共治共享的社会治理格局基本形成。

做好当前和今后一个时期政府工作，要坚持以党的建设高质量推动经济发展高质量这一工作方向，把握好以下三个方面：一是始终坚持以习近平新时代中国特色社会主义思想为指导，深入贯彻习近平总书记调研指导河南时的重要讲话精神，坚定落实中央和省委、省政府决策部署，牢记嘱托，不辱使命，助力中原更加出彩。二是落实市委战略部署，选准坐标和参照系，围绕建设重要区域中心城市目标定位，强力实施“两轮两翼”战略，重抓重推“九大专项”，全面提升区域整体发展水平，奋力争当区域振兴领跑者。三是完善政府工作思路举措。坚持党建是最大政绩、发展是第一要务、稳定是第一责任、人才是第一资源、创新是第一动力，坚持民生优先、生态优先、农村优先，坚持城乡融合、三产融合、三农融合，坚持以人为核心、培育壮大人才队伍，坚持目标导向、任务导向、问题导向，坚持政府主导、市场引导、规划引领、项目支撑，发挥好政策投资、企业扩张、回归创业、全民创业、招商引资“五个主体”作用，实现搞活存量、优化增量、扩充总量、提升质量“四量统筹”。

2018年是贯彻党的十九大精神的开局之年，是改革开放40周年，是决胜全面建成小康社会的关键一年，也是新一届政府的起步之年，我们要全力以赴做好今年各项工作。综合考虑发展形势和工作导向，建议2018年全市经济社会发展主要预期目标为：生产总值增长7.5%以上，规模以上工业增加值增长8%以上，固定资产投资增长9%左右，社会消费品零售总额增长11%以上，进出口保持平稳增长，一般公共预算收入增长6.5%以上，税收占比明显提升，居民人均可支配收入高于经济增速，城镇新增就业5.25万人，城镇调查失业率、登记失业率分别控制在5.5%以内和4.5%以内，城镇化率提高1.5个百分点以上，居民消费价格涨幅控制在3%左右，内乡、镇平、方城3个县脱贫摘帽，14.54万农村贫困人口稳定脱贫，完成1.49万贫困人口易地扶贫搬迁，环境质量持续改善，节能减排、环境保护等指标完成省下达的目标任务，供给侧结构性改革取得实质性进展，发展质量效益持续提高，人民群众获得感不断增强。

重点抓好6个方面工作：

（一）深化“两轮两翼”战略，夯实高质量发展的战略支撑

“两轮两翼”战略，符合上级精神，符合南阳实际，充满生机活力，是统筹各方、统揽全局、推动高质量发展的重要载体和抓手，必须坚定不移、坚持不懈，聚焦聚力、始终如一。

1.强化项目建设支撑。深化“项目建设突破年”活动，系统谋划，强力推进，实现项目提质、提量、提速新突破。突出重点。围绕“九大专项”，聚焦实体经济项目、工业项目、高新技术项目，以产业补链延链强链、强基础补短板等项目为方向，实施3000万元以上固定资产投资项目1396个，突出抓好省、市重点项目和“三个三十”重大项目，推动项目建设滚动接续发展，确保省、市重点项目投资增长20%以上。拓展投资渠道。把争取上级投资作为重中之重，组建专业团队，全面精准对接，抢抓中央新一轮扩投资、补短板的重大机遇，加大政府债券争取力度，深入谋划推进一批基础性、战略性项目。支持现有企业扩张，通过上市（挂牌）、战略重组等方式，不断发展壮大。重视回归人员创业，加强引导，大造声势，发展回归经济。推进大众创业、万众创新，提供全方位服务，建设“双创”示范基地，培育孵化一批优质企业和项目，打造“双创”升级版。抓好招商引资引智，选择有实力、有诚意、有业绩的企业，对本地企业和外来投资主体，一视同仁，平等对待。务求实效。围绕“快、优、省、廉”，强化工作组织，上下联动、条块结合，领导分包、专班推进。强化要素保障，提升审批效率，解决项目前期和用地、融资等突出问题。强化督导考核，实现统计数字、客观形象、主观印象相一致，推动项目建设“平地起谷堆”。

2.强化科技创新支撑。把创新摆在发展全局的突出位置，抓好“四个一批”，促进“四个融合”，激活高质量发展的第一动力。培育创新主体。强化企业主体地位，加快培育创新引领型企业，培植、扶持科技创新百强企业和纳税百强企业，新增高新技术企业10家，实施10个市级重大科技专项。搭建创新平台。加大投入、完善条件，加快建设科技创新平台，新培育市级以上创新平台40家。加快成果转化。推进科技大市场规范高效运行，发布、推广和转化一批成熟科技成果。深化科技金融管理创新，建好用好科技成果转化基金，组织实施一批“科技保”、“科技贷”和知识产权质押贷款项目。推进开放创新。积极开展市厅、市校、市院合作，深化与中科院、省科技厅、省农科院、省科学院等合作，争取省级以上科技项目50个。支持我市企业与省内外高校、科研院所开展科技交流合作。抓好与中关村的合作，加快中关村南阳科技园建设，打造中关村南阳协同发展创新中心。强化政策支持。抓住郑洛新国家自创区“一顶帽子大家戴”的政策机遇，以高新区为主阵地，承接好政策红利。不断加大财政投入，扩大科技创新贷款贴息和创新券规模。

3.强化现代金融支撑。重视金融、研究金融、操作金融、搞活金融，实现以土地为核心经营城市、金融与产业之间、企业层面三个链条的良性循环，为高质量发展提供有力支撑。发展金融产业。加快示范区金融街二期建设，鼓励引导金融机构入驻。对接引进兴业银行、恒丰银行等金融机构，完成农信社改制组建农商行任务。推动银行、证券、保险、基金等各类金融机构协同发展，大力发展普惠金融、科技金融、绿色金融，积极申报创建绿色金融改革创新试验区。用好金融工具。加快投融资平台改革发展，优化平台资源，创新体制机制，推进市场化改革，构建省、市联动的投融资机制，提升自我发展能力。用好PPP模式，特别是加强政策性、开发性金融争取工作，重点推进内河治理、国家储备林、棚户区改造等一批基础设施和重点项目建设。推进财政涉企资金基金化改革，整合优化本土基金，积极引进市外基金，参与省级基金设立，引导、撬动各类社会资本支持我市经济发展。服务实体经济。推进企业上市，抓住贫困县企业上市绿色通道机遇，实行市级统筹，加大扶持力度，推进金冠电气、想念食品、仲景大厨房进入上市辅导，30家企业在中原股交中心挂牌。扩大信贷投放，做强政府性担保平台，扩大转贷资金池规模，完善落实对金融机构的考核奖励办法，实现信贷投放增长10%以上。

4.强化人力资源支撑。实施“诸葛英才计划”，以党政人才、企业经营管理人才、专业技术人才、高技能人才为重点，统筹抓好各类人才队伍建设。引进人才。围绕主导产业，突出“高精尖缺”，引进一批领军人才。加强驻外人才工作站建设，吸引各类人才回乡创业就业。实行柔性引才，不求所有、但求所用。培养人才。依托国家、省重大人才支持项目，培养一批高层次人才和创新创业团队。推进全民技能振兴，深化职教攻坚，培养一批专业技术人员和熟练技能人才。重视企业家队伍建设，关心、支持、鼓励、帮助企业家，培育百名科技企业家和百名青年企业家。用好人才。解放思想、更新观念，改革体制、创新机制，广纳贤良、广聚英才，完善人才评价体系，深化职称制度改革，赋予用人单位更多自主权，鼓励支持科研人员兼职科技服务或离岗创业。落实政策，优化服务，优化环境，建立“一站式”服务平台，实施人才安居工程，让各类人才安心工作、专心创业、舒心生活，实现留住人才、人尽其才、才尽其用、用有所成。

（二）推进产业结构优化升级，构筑高质量发展的重要基石

持续推进转型发展攻坚，以建设先进制造业强市、现代服务业强市、现代农业强市为目标，以高端化、绿色化、智能化、融合化为方向，落实“破、立、降”要求，推动产能、产业、产品结构调整，优化三次产业结构，加快构建现代产业体系。

1.抓重点产业。培育“千百亿产业集群”，发展“一县一业”，重点培育装备制造、绿色食品、纺织服装三个千亿级产业。围绕建设先进制造业强市，重点发展智能装备制造、生物医药、智能终端、新能源汽车、大数据、软件开发6个新兴产业，改造提升装备制造、汽车及零部件、食品加工、纺织服装、化工、建材6个传统产业，巩固提升烟、酒、油、电、碱5个高税利产业。推进“三大改造”，创建2家~3家省级绿色工厂，建设16个智能化车间（工厂），重点实施95个技术改造项目。围绕建设现代服务业强市，重点发展文化旅游、健康养生、现代物流、现代金融、电子商务、高端航空服务6个产业。围绕建设现代农业强市，推动三产融合、三农融合，重点发展都市生态农业、绿色有机农业、现代畜牧业，着力培育香菇、猕猴桃、月季、艾草等特色产业，加快发展农业生产性服务业。

2.抓重点区域。加快产业集聚区提质转型发展，推动主导产业精准招商、产品技术工艺创新、落后产能“腾笼换鸟”，盘活闲置土地、厂房等要素资源，推动产业集聚区、服务业“两区”提质升级，支持引导产业集聚发展。培育科技示范园区、田园综合体、农业产业化集群等现代农业发展载体。

3.抓重点企业。开展“双百企业”培育，实施一个路径、一个规划、一个目标、一个战略合作伙伴的发展举措，因企制宜，精准规划，精准指导，持续开展好“四项对接”和降成本专项行动，落实首席服务员制度，推动100家骨干企业做大做强，100家“专精特新”企业发展壮大，新增销售收入50亿元以上企业2家、30亿元以上企业3家、10亿元以上企业4家。

4.抓重点项目。加快实施一批大项目、好项目，以优质增量引领产业转型升级。制造业方面，谋划实施185个重点项目，强力推进星凯龙新能源汽车、防爆电气产业园、利达光电超硬功能薄膜生产基地、荣阳实业搬迁、机器人生产、乐凯华光印刷电子新材料产业园、印刷线路板、5G高频电子板、中光学投影机生产基地、杭萧钢构产业园等重大项目。服务业方面，滚动实施100个重点项目，抓好老界岭创建国家级旅游度假区、张仲景健康城、河南万邦物流、跨境电商产业园等重大项目。农业方面，加快推进新野肉牛产业化集群创建国家三产融合先导区、中以高效农业科技示范园、邓州杂交小麦基地等项目。

5.抓重点品牌。深入开展“质量提升年”活动，持续推进制造业增品种、提品质、创品牌，努力建设先进制造业中心。培育服务业知名品牌，打造全国知名生态文化旅游目的地、全国健康养生之都、现代金融中心、现代物流中心、现代商贸中心。推进标准化生产，开展“三品一标”认证，强化农产品质量安全监管，扩大农产品进京规模，进一步打响“中线渠首”绿色有机农产品品牌。

（三）统筹城乡协调发展，优化高质量发展的区域布局

坚持以人为核心、城乡融合、三产融合、三农融合，统筹中心城区振兴工程和乡村振兴战略，推进百城建设提质工程，推动城乡协调发展、互促共进。

1.推进中心城区振兴工程。中心城区是建设重要区域中心城市的龙头和关键。要突出高水平规划、高标准建设、高效率管理，坚持硬件与软件、新区与老区、地上与地下、宜业与宜居“四个统一”，做好以水润城、以绿荫城、以文化城、以业兴城“四篇文章”，持续抓好“双创双建”，推动中心城区加速振兴崛起。加快新城区建设。突出“满城绿色半城水”，注重功能完善和产业布局，全力以赴把新城区建设好。抓住世界月季洲际大会举办和高铁开通两个关键节点，细化任务，倒排工期，加快推进高铁片区、世界月季洲际大会配套工程、水系景观等22个重点项目。抓好老城区提升。强力推进卧龙岗武侯祠创建国家5A级景区和古宛城保护利用两个标志性项目。完成梅溪河、三里河治理任务，解决好温凉河、汉城河遗留问题，全面加快推进十二里河、溧河治理工程。新建、改造城市道路25条、城市供气管网186公里、供热管网14公里。抓好公园游园、河道绿化、道路绿化等工程建设，积极创建国家生态园林城市。改造背街小巷和老旧小区。在学校、医院、车站等重点区域，谋划建设过街天桥和地下通道。创建公交都市，推广新能源公交车，对城乡客运班线进行公交化改造，推进公交场站和公共停车场建设。

2.加快县城发展。统筹实施百城建设提质工程和文明城市创建，不断提高县城综合承载能力，打造生态宜居、充满活力、各具特色的现代化城市。依托产业集聚区和服务业“两区”，培育特色主导产业，强化产业支撑，推动产城融合。研究实施加快“一心六组团”发展的政策措施，推进中心城区至组团城市间快速通道建设，启动南阳至社旗、南阳至唐河、南阳至官庄、南阳至鸭河轨道交通规划研究，逐步实现规划一体、交通连接、产业配套、功能互补。提升邓州副中心城市功能，推动县市区形成各具特色、竞相发展、多点支撑的良好局面。抓好50个重点镇，培育一批5万~10万人口的中心镇、特色镇。

3.实施乡村振兴战略。坚持农业农村优先发展，按照产业兴旺、生态宜居、乡风文明、治理有效、生活富裕的总要求，用市场经济、工业和城市的理念、方法审视领导农业农村工作，推动实现农业强、农村美、农民富。夯实产业基础。深化农业供给侧结构性改革，重生态、重质量、重品牌，加快由增产导向转变为提质导向，稳步提升粮食综合生产能力，着力推进“四优四化”。新发展绿色有机农产品生产基地20万亩,新认证绿色有机产品50个。发展畜产品、面制品、食用菌等精深加工产业，实施想念食品工业园等一批重点项目。推进农村三产融合发展，发展农村电商物流，培育壮大农业产业化集群，加快田园综合体建设，大力发展乡村旅游。发挥农民主体作用。坚持尊重农民意愿、维护农民利益，充分发挥农民投身乡村振兴的积极性、主动性和创造性。拓宽增收渠道，千方百计增加农民经营性、财产性、工资性收入。创新生产经营方式、社会服务体系、产业组织模式，提高农民组织化程度，大力培育新型农业经营主体，发展多种形式适度规模经营，推动小农户和大生产、大市场有机衔接，新发展农民专业合作社1000家、家庭农场500家。有组织地开展农村劳动力转移就业，新增1.5万人返乡下乡创业。加强乡村治理。夯实农村基层组织，打造一支懂农业、爱农村、爱农民的“三农”工作队伍。完善乡村矛盾调解和便民服务机制，深入推广“四议两公开”，健全自治、法治、德治相结合的乡村治理体系，严厉打击“村霸”和宗族恶势力，决不允许其横行乡里、欺压百姓、侵蚀基层政权。加强农村精神文明建设，推动移风易俗。抓好垃圾处理、污水治理、厕所革命、农业生产废弃物资源化利用、村容村貌整治提升五项重点工作，开展“四美乡村”创建活动，完善农村基础设施和公共服务，推进农村能源改革和信息进村入户工程，新建农村公路700公里，完成农村危房改造2.78万户，新建、改建乡镇、中心村公厕360座以上。

4.完善体制机制。强化规划引领，完善提升各级各类规划，启动新一轮城市总体规划修编，编制新城区详细规划、城市设计和乡村振兴战略规划，实施农村人居环境整治、乡风文明建设、乡村治理体系等领域三年行动计划。加强土地管理。坚持“六统一”，加大土地收储力度，持续规范提升中心城区房地产市场。破解融资难题。研究落实搞活资源、资产、资本的措施，紧跟国家政策节拍，多措并举解决城乡发展资金问题。建立城乡融合发展机制。坚持基础设施建设向农村倾斜、公共服务在农村优先发展，推动人才、资金、技术下乡，推动城乡要素双向流动，推动农村人口有序流动、产业有序集聚；深化农村改革，加快承包地、宅基地“三权分置”和农村集体产权制度改革，积极推动农村产权综合交易平台建设，实现资源变资产、资金变股金、农民变股东，为乡村振兴注入活力。

5.推进基础能力建设。优化现代交通、信息、能源、水利设施网络，推动局部优势、单一优势向综合竞争优势转化，为高质量发展蓄积更大势能。交通方面，围绕构建现代综合交通枢纽，完成南阳火车站改扩建工程，加快推进郑万高铁、蒙华铁路建设，积极做好宁西高铁、呼南高铁豫西通道、南阳机场迁建争取和前期工作；开展旅游轨道交通环线前期规划研究；加快周南、栾西高速公路建设，推进西十、方枣高速公路前期工作，谋划打造全市高速旅游环线；积极推进唐河航运工程。信息化方面，以满足社会治理和公共服务需要为重点，统筹规划、整合资源，加快建设智慧城市；推进“全光网南阳”全面升级，完成“宽带中国”示范城市建设；发展大数据产业，加快建设白河大数据产业园。能源方面，重点推进内乡煤电运一体化、南召天池抽水蓄能电站等重大项目，大力发展风电、光伏发电、生物质能，积极申报光伏发电领跑基地项目，开工建设垃圾焚烧发电项目。水利方面，重点推进方城罗汉山等大中型水库和唐白河、丹江防洪治理工程，加快实施大中型灌区续建配套节水改造、农村饮水安全巩固提升等工程，积极争取丹江口库区上游水土保持项目。强力推进自备井封停和置换南水北调水源工作，年底前让中心城区群众喝上丹江水。

（四）全面深化改革开放，激发高质量发展的动力活力

以改革开放40周年为重要契机，持续深化改革，不断扩大开放，走好高质量发展的必由之路。

1.聚焦重点改革。坚持“一跟进、两聚焦”，高标准推进各项改革。推进政府职能转变和“放管服”改革。全面推行市场准入负面清单制度，积极推进“证照分离”试点改革。进一步精简行政审批事项，清理各类“奇葩证明”，没有法律法规依据的一律取消。强化市场监管，全面推行“双随机、一公开”监管模式，构建以信用承诺、联合惩戒、信息公示为核心的信用监管体系，让守信者一路畅通，失信者寸步难行。创建全国社会信用体系建设示范城市。推进公共资源一个平台交易，实现应进必进。推进政府机构改革。按照省委、市委的统一部署，提高认识、统一思想，精心组织、有条不紊，推进政府机构改革，确保思想稳定、工作稳定、队伍稳定、大局稳定。推进事业单位、国有企业公务用车改革。推进经济领域改革。分类推进国有企业改革，由国有工业企业向国有非工业企业拓展，持续推进产权多元化和混合所有制改革，剥离企业办社会职能，抓好“僵尸企业”处置，妥善解决遗留问题。深化财税和金融体制改革，认真落实预算管理体制和税制改革，全面实施绩效管理，使财政资金花得其所、用得安全。稳妥推进经营类事业单位改革。实施河道砂石资源管理国有化改革。推进民生领域改革。深化医药卫生体制改革，加快推进公立医院综合改革，实施医疗、医保、医药联动，下大力气解决群众看病就医难题。持续深化教育、科技、文化、体育、价格等领域改革。

2.提升开放水平。拓展开放范围和层次，完善开放布局和体制机制，以高水平开放推动高质量发展。提升开放平台。完善提升综保区功能，抓好招商运营。推进跨境电子商务综合试验区建设。精心筹备好世界月季洲际大会。加快外贸转型升级基地建设，培育外贸进出口企业，扩大香菇等优势产品出口，大力发展外向型经济。扩大开放合作。积极主动开展京宛对口协作，坚持引领互动、加强宣传，远近结合、全面深化、精准对接，与京津冀地区建立全方位合作，力争在高端产业、创新创业、现代金融、教育卫生、文化旅游、健康养生、生态农业等方面实现突破。全面对接中国（河南）自由贸易试验区，积极融入郑州航空港经济综合实验区。主动融入长江经济带，积极参与“一带一路”建设，加强与长三角、珠三角的合作，承接产业集群转移。提高招商实效。坚持招大招强、走出去和请进来相结合、引资与引智引技相结合。围绕“九大专项”，实行定点对接、定向招商、集群引进，组织好承接北京非首都功能疏解等系列招商活动。夯实招商机构建设，完善社会资本承接机制，开展市场化招商，探索中心城区招商一体化管理，推动招商项目落地见效。优化营商环境。把营商环境作为发展的“金字招牌”，研究探索制定优化营商环境工作条例，打造便捷高效的政务环境、更具活力的创新创业环境、宽松有序的企业经营环境、竞争高效的市场环境、公平公正的法治环境、重商安商的社会环境，让南阳成为投资的洼地、企业成长的沃土。把深化“放管服”改革作为优化营商环境的重中之重，强力推进、持续深化“一网通办”前提下的“最多跑一次”改革，让数据多跑路，让群众和企业少跑腿。构建亲清新型政商关系，制定政商交往“正面清单”和“负面清单”，着力解决“清而不亲”的问题，倾听企业家心声，千方百计为企业办好事、解难事。弘扬企业家精神，培育企业家文化，给予优秀企业家应有的政治荣誉，让遵纪守法、创业奉献的企业家有舞台、有地位、受尊重。落实支持非公有制经济发展的政策措施，鼓励更多个体工商户转变为企业、更多小微企业升级为规模以上企业。

（五）打好三大攻坚战，补齐高质量发展的短板

全力以赴打好防范化解重大风险、精准脱贫、污染防治三大攻坚战，为决战决胜全面建成小康社会打下坚实基础。

1.打好防范化解重大风险攻坚战。要强化底线思维，坚持问题导向、标本兼治，努力营造安全稳定的发展环境。防范化解金融风险。落实监管责任，严厉打击非法集资，整顿小贷担保公司，开展互联网金融专项整治，规范发展政府投融资平台。全面防控社会风险。树立“万无一失、一失万无”的理念，扛牢反恐维稳、水质保护政治责任。完善排危除险机制，加强防灾减灾救灾体系建设。严格落实安全生产责任制，守住不发生重特大安全事故的底线。强化社会治安综合治理，推进治安防控“雪亮工程”，依法做好信访工作，加强舆情引导管控，确保社会大局和谐稳定。强力推进扫黑除恶专项斗争，全力以赴、尽锐出战，依法严惩，扫黑务净、除恶务尽，斩草除根、铲除土壤，坚决清除各类黑恶势力，坚决打掉背后“保护伞”，决不让黑恶势力有任何生存空间。坚持打、防、建相结合，建立完善扫黑除恶的长效体制机制，给人民群众长治久安的安全感。

2.打好精准脱贫攻坚战。要感恩时代，珍惜机遇，增强责任感、紧迫感、积极性、主动性、全局意识和大局意识，无私奉献，无怨无悔。思想上更加重视，工作上更加用心，作风上更加扎实，坚持以脱贫攻坚统揽经济社会发展全局，统筹谋划，深度融合，精准衔接，一个季度突出一个主题，全面提升脱贫质量，让脱贫得到群众认可、经得起历史检验。全面精准施策。打好产业扶贫、就业创业扶贫、生态扶贫、金融扶贫“四场硬仗”，开展健康扶贫、教育扶贫、扶贫助残、易地扶贫搬迁、危房改造清零、扶贫扶志“六项行动”，实施交通扶贫、饮水安全、电网升级、环境整治“四大工程”，主攻产业就业扶贫，培育龙头企业，发展特色产业，加强技能培训，大力推广牧原“3+N”扶贫模式，促进贫困地区贫困群众稳定脱贫、可持续发展。突出重点难点。聚焦深度贫困，把深度贫困县淅川和全市199个深度贫困村作为重中之重，强化教育、交通、医疗、水利、电力等专项扶贫，改善生产生活条件。聚焦重点人群，把鳏寡孤独、特困供养对象、重度残疾人员作为重点，落实好医保救助工程等措施，做实政策兜底。特别是对智障贫困人口，探索实行以乡、村为单位的集中供养。注重统筹抓好非贫困县、非贫困村脱贫攻坚工作。激发内生动力。把扶贫和扶志扶智结合起来，理直气壮、深入细致做好群众工作，建立正向激励机制，宣传表彰脱贫致富先进典型，丰富群众精神文化生活，倡导勤劳、节俭、知足、感恩的社会风尚，提振贫困群众精气神。侯卫东官场笔记

3.打好污染防治攻坚战。牢固树立绿水青山就是金山银山的发展理念，坚决打赢蓝天、碧水、净土保卫战，抓住中央环保督察“回头看”反馈问题整改的机遇，遏制污染增量，消除污染存量，加强生态环境保护和修复，努力实现人与自然和谐发展，守住这片祖先赐给我们的天生丽质的风水宝地。深入推进大气污染防治攻坚战。坚持标本兼治、综合施策，打好结构调整优化、工业企业绿色升级、柴油货车治理、城乡扬尘全面清洁、环境质量监控全覆盖5个标志性攻坚战，确保大气环境质量持续改善，努力让全市人民享有更多蓝天白云。持续推进水污染防治攻坚战。认真落实河长制湖长制，全面贯彻《白河水系水环境保护条例》，打好黑臭水体治理、饮用水源地整治、全域清河行动、农村环境治理等4个标志性攻坚战，确保出境水稳定达标，南水北调中线水源地水质稳定保持在Ⅱ类以上。启动土壤污染防治攻坚战。实施农用地土壤环境分类管理，开展土壤污染治理与修复，加强农村农业面源污染防治，严格危险废物环境监管，加强生活污染源管控。加强生态系统保护和修复。坚持节约优先、保护优先、自然恢复为主，划定生态保护红线，加快森林、湿地、流域、农田、城市五大生态体系建设，推进海绵城市建设和城市“双修”，扎实开展大造林大绿化，加快推进国家储备林、白河国家湿地公园等重点项目，谋划建设伏牛山世界植物大观园，完成营造林164万亩。加强生态文明制度建设，建立健全市场化、多元化生态补偿和生态环境损害赔偿制度。加快实施污染物排放许可制度和排污权交易制度。

（六）保障和改善民生，落实高质量发展的根本目的

牢固树立以人民为中心的发展思想，把群众关切作为努力方向，把群众满意作为工作标准，尽力而为、量力而行，多办利民实事，多解民生难事，兜牢民生底线，不断提升人民群众的获得感、幸福感、安全感。

1.提高人民群众收入水平和就业质量。把增加居民收入作为检验发展质量和改善民生的重要标准，确保居民收入增速不低于经济增速。完善就业创业服务体系，促进高校毕业生、农民工、城镇就业困难人员、退役军人等重点群体多渠道就业创业，健全劳动关系协商机制，实现更加公平、更加充分、更高质量就业。依法治理、坚决解决拖欠农民工工资问题。

2.办好人民满意的教育。百年大计，教育为本。教育是国之大计、党之大计。落实教育优先发展战略，在经济社会发展规划上优先安排教育，在财政资金投入上优先保障教育，在公共资源配置上优先满足教育，在全社会形成尊师重教的浓厚氛围。实施第三期学前教育行动计划。推动城乡义务教育一体化均衡发展，教育投入继续向农村等困难地区和薄弱环节倾斜。实施好“全面改薄”和扩充城镇义务教育资源五年计划。改善540所农村义务教育薄弱学校办学条件，完成154所农村义务教育寄宿制学校建设，优化农村中小学师资队伍，办好农民家门口的义务教育。加快推进中心城区15年一贯制完全学校建设，第一批年底前全部竣工，第二批年底前全面开工，第三批年底前完成选址和设计；抓好中心城区6所中小学新建改扩建工程，新增学位1.1万个；统筹整合闲置中专、技校、干校和培训中心等资源，用于加强基础教育。全力以赴解决“大班额”、“入学难”、“择校热”等群众反映强烈的问题。推进普及高中阶段教育。优化整合职业教育资源，加快推进职教园区建设。支持高等教育发展，优化办学环境，加快硕士点建设，全面提升办学水平。实施第二期特殊教育提升计划。支持和规范社会力量兴办教育。健全学生资助制度，努力让每个孩子享有公平而有质量的教育。

3.健全社会保障体系。全面实施全民参保计划。积极参与养老保险全国统筹，继续提高退休人员基本养老金和城乡居民基础养老金标准。推动城乡居民基本医保与大病保险制度有效衔接，城乡居民基本医保财政补助提高到每人每年490元，巩固、深化、提升医保救助工程。健全社会救助体系，支持公益慈善事业发展，稳步提高城乡低保、社会救助、抚恤优待标准。健全农村留守儿童、妇女、老年人关爱服务体系。实施好“政福保”工程，让遭遇突发性事故的群众能得到普遍救助。

4.努力解决群众住房问题。规范发展房地产业，稳定商品住房销售价格，探索建立多主体供给、多渠道保障、租购并举的住房制度。落实新的三年棚改计划，今年基本建成1万套以上。加大公租房保障力度，解决住房困难家庭基本住房需求。提升社区物业管理水平。妥善解决农运会涉迁群众安置问题，努力让广大群众实现安居宜居。

5.建设健康南阳。弘扬尊医重卫的社会风尚，加快医疗卫生事业发展，优化资源布局，规划建设市中医院、中心医院新院区，推动优质医疗资源下沉，加强基层乡村医疗卫生机构标准化建设。推进分级诊疗、家庭医生签约服务和医联体建设，加强全科医生队伍建设，逐步解决看病难、看病贵问题。提高食品药品安全水平，让群众吃得更放心、用药更安全。开展形式多样、丰富多彩的全民健身活动。发挥特色优势，打响医圣仲景品牌，强力推进医圣祠、新城区、紫山张仲景健康文化园、健康产业园、健康养生园建设，谋划推进智慧康养产业发展，实现教育、医疗、养生养老融合发展，打造健康之城、养生之都。

6.发展文化事业和文化产业。培育和践行社会主义核心价值观，大力弘扬焦裕禄精神、红旗渠精神、愚公移山精神、南水北调精神，开展公民思想道德素质、诚信守法、文明风尚提升行动。实施文化惠民工程，加强基层综合文化服务中心建设，深入开展“唱响白河”等群众文化活动。加强网络建设治理，营造清朗网络空间。启动编制古城、古镇、古村落、古建筑保护规划，开展文物安全集中整治，加强文化遗产保护与传承。创新发展文化产业，培育一批文化产业园区、骨干企业和知名品牌。大力发展玉文化产业。推进文化产业与生态旅游融合发展，培育龙头景区，发展全域旅游，持续打造“避暑养生地、南阳老界岭”旅游品牌，建设国内知名生态文化旅游目的地。

加强国防教育和国防后备力量建设，支持驻宛人民解放军、武警部队、民兵预备役和人民防空建设，持续开展双拥工作，推进军民融合发展，巩固军政军民团结。广泛开展法治宣传教育，完善公共法律服务体系，做好法律援助和社区矫正工作。认真做好第四次全国经济普查工作。支持工会、共青团、妇联、工商联、残联等人民团体更好发挥桥梁纽带作用。加强外事、侨务、民族、宗教、对台、史志、档案、地震、援疆等工作。

三、加强政府自身建设

推动高质量发展，建设重要区域中心城市，政府重任在肩、责无旁贷，必须不忘初心，牢记使命，继续奋斗，再立新功。我们将围绕一个“新”字：新时代、新起点、新征程，要有新气象、新作为；突出一个“干”字：想干、会干、实干、正干。坚持以民为本、以实为魂、以干为乐、以廉为荣，坚持出于公心、善于用心、令人放心，始终保持焦裕禄精神的“三股劲”，做到忠诚干净担当、为民务实清廉，建设人民满意的服务型政府。

1.加强政治建设。旗帜鲜明讲政治，牢固树立“四个意识”，自觉践行“两个坚决维护”，增强“四个自信”，做到“四个服从”，严明政治纪律和政治规矩，思想上高度认同、政治上坚决维护、组织上自觉服从、行动上紧紧跟随，在政治立场、政治方向、政治原则、政治道路上，始终同以习近平同志为核心的党中央保持高度一致，坚持党的领导，自觉接受市委领导，落实市委决定，贯彻落实市委意图，全面维护市委形象。推进“两学一做”学习教育常态化制度化，认真开展“不忘初心，牢记使命”主题教育活动。

2.坚持依法行政。牢固树立法治理念，严格遵守宪法法律，加快建设法治政府，把政府工作全面纳入法治轨道。推动科学、民主、依法决策，确保政府的每一项决策都遵从法律法规、体现人民意愿。坚持严格规范公正文明执法，切实维护公平正义。依法接受市人大及其常委会的监督，自觉接受市政协的民主监督，坚决执行人大及其常委会的决议决定，认真办理人大代表建议和政协提案，广泛听取民主党派、工商联、无党派人士、人民团体和社会各界人士意见。主动接受监察监督、审计监督、社会监督、舆论监督。推进政务公开，让权力在阳光下运行。

3.提高政府效能。以“放管服”改革和作风建设为抓手，转变政府职能，提高政府效能。严格落实中央八项规定及实施细则精神和省、市办法，驰而不息纠正“四风”。认真开展“干部作风整顿年”活动，聚焦执政为民和决策落实，旗帜鲜明给积极干事者撑腰鼓劲、对懒政怠政者严肃问责，绝不鼓励和培养懒汉、懦夫，决不能表态多调门高、行动少落实差，决不允许占着位子不干事。

4.强化担当作为。要想干，坚持“公”字当头。立党为公，执政为民，以人民为中心，人民政府为人民，牢记党的宗旨意识和群众路线，强化事业心、责任感、使命感、危机感，珍惜机遇，珍惜岗位，无怨无悔，任劳任怨，只争朝夕，勇往直前，无负无愧，做到出于公心，一心为公，克己奉公，大公无私，公而忘私。要会干，坚持“学”字当头，以干为乐。想清悟透，学思践悟，学政治、学理论、学业务，提高政治业务素质和说写干能力。要学会使用望远镜和显微镜，把握好宏中微观，当好规划师、设计师、工程师、工匠技师，努力成为行家里手。要善于把握形势规律，明确目标任务，统筹谋划设计，讲究工作方法，完善赏罚机制，调动各方面的积极性，营造健康的政治生态，树立工作导向，使工作有张有弛、有板有眼、有条不紊。要实干，坚持“实”字当头，以实为魂。实事求是、尽力而为、量力而行，“跳起来摘桃子”,说实话、办实事、出实招，任劳任怨、埋头苦干，只干不说、多干少说、先干后说、干成再说。目标要实、规划要实、措施要实，解放思想、更新观念、与时俱进，大胆改革开放、探索创新，高标准、快节奏、重实效，比发展、争位次、创一流，做到出工、出力、出效、出彩。要正干，坚持“廉”字当头，以廉为荣。清正廉洁，讲政治，讲大局，讲党性，行得正，站得直，走正路，正己正人，以身作则，严格要求，遵纪守规。落实全面从严治党主体责任和“一岗双责”，深入推进政府系统党的建设和党风廉政建设，运用好监督执纪“四种形态”，坚决惩治各类腐败行为，巩固发展反腐败斗争压倒性态势。要善于抓班子、带队伍，看好自己的门，管好自己的人；干成事，不出事，依法办事，提高效率，做到“快优省廉”；营造风清气正、公道正派、公平公正的环境，逐步形成不敢腐、不能腐、不想腐的机制。

各位代表！新时代要有新气象，新征程要有新作为。南阳振兴、匹夫有责，机不可失、时不我待，一万年太久、只争朝夕。让我们高举习近平新时代中国特色社会主义思想伟大旗帜，在市委的坚强领导下，不忘初心、牢记使命，锐意进取、埋头苦干，团结带领全市人民，全力推动高质量发展，加快建设重要区域中心城市，为决胜全面建成小康社会、谱写中原更加出彩的南阳篇章而努力奋斗！
